# Vascular optimality dictates plant morphology away from Leonardo’s rule

**DOI:** 10.1073/pnas.2215047120

**Published:** 2023-09-18

**Authors:** S. B. D. Sopp, R. Valbuena

**Affiliations:** ^a^School of Natural Sciences, Bangor University, Bangor LL57 2UW, United Kingdom; ^b^Division of Remote Sensing of Forests, Swedish University of Agricultural Sciences, Umeå SE-901 83, Sweden

**Keywords:** metabolic scaling theory, plant science, ecology

## Abstract

Metabolic scaling theory (MST) provides an understanding of scaling in organismal morphology. Empirical data on the apparently universal pattern of tip-to-base conduit widening across vascular plants motivate a set of generalized MST (gMST) relationships allowing for variable rates of conduit coalescence and taper and a transition between transport and diffusive domains. Our model, with coalescence limited to the distalmost part of the conductive system, reconciles previous MST-based models and extends their applicability to the entire plant. We derive an inverse relationship between stem volume taper and conduit widening, which implies that plant morphology is dictated by vascular optimality and not the assumption of constant sapwood area across all branching levels, contradicting Leonardo’s rule. Thus, energy efficiency controls conduit coalescence rate, lowering the carbon cost needed to sustain the vascular network. Our model shows that as a plant grows taller, it must increase conduit widening and coalescence, which may make it more vulnerable to drought. We calculated how our gMST model implies a lower carbon cost to sustain a similar network compared to previous MST-based models. We also show that gMST predicts the cross-sectional area of vessels and their frequency along the relative length better than previous MST models for a range of plant types. We encourage further research obtaining data that would allow testing other gMST predictions that remain unconfirmed empirically, such as conduit coalescence rate in stems. The premise of energy efficiency can potentially become instrumental to our understanding of plant carbon allocation.

## Significance

We outline a model that generalizes allometric scaling theory to the entire plant by transitioning between distinct vascular domains. We show how the entire network can be optimized based solely on the maintenance of hydraulic resistance, by adjusting the rate of conduit widening and coalescence. This economizes the carbon employed in developing an energy-efficient vascular system that combines transport and diffusive functions. We deduce an inverse relationship between the widening of conduits tip to base and the tapering of branch volume base to tip. This relationship contradicts the largely accepted Leonardo’s rule that the combined area of stems is preserved along the length of the plant. Our postulates may explain the greater vulnerability of large trees to climate change.

The exact nature of biological scaling relationships has been argued about for centuries, typically cited as beginning with Leonardo da Vinci who first proposed that total sapwood area is constant at all levels of branching, such that the summed areas of sapwood in the terminal twigs is the same as the sapwood area at the base. Many biological models have since taken inspiration from Leonardo’s rule to model both plant exterior branching networks and their vascular systems, despite there being little evidence of the rule occurring consistently (1). Metabolic scaling theory (MST) (2–4) is the leading theory of explanation on organism form, utilizing principles of area preservation for both the external and vascular branching networks within plants.

**Fig. 1.**
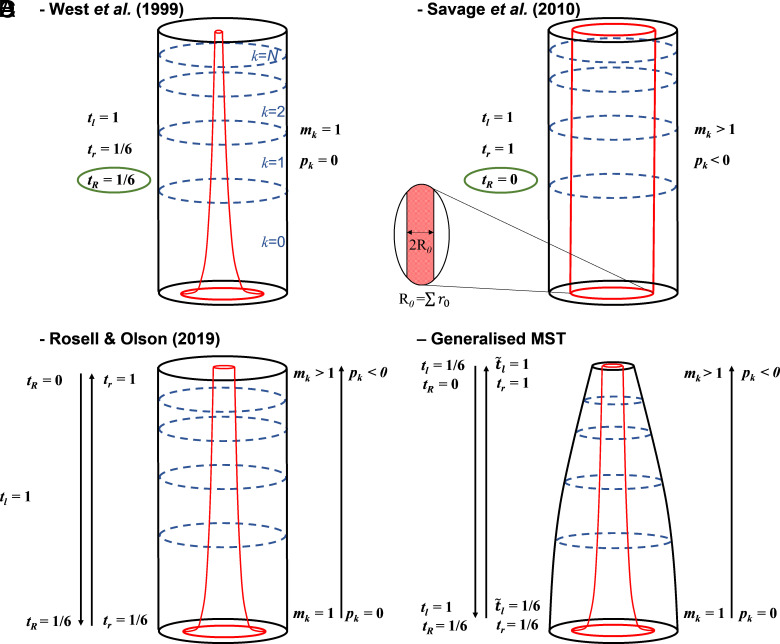
Models of summated branching network volumes, illustrating how total network volume (outer shape in black) and total conductive volume (inner shape in red) change with varying tapering coefficients ($t_{R}$, $t_{r}$ and $t_{l}$), and coalescence rates ($m_{k}$ and $p_{k}$) (Eqs. **1**–**7**). Four network models are presented, showing the differences between (*A*) West et al. (3), (*B*) Savage et al. (4), (*C*) Rosell and Olson (8) and (*D*) our gMST relationships of plant morphology, reaching from the plant base to the tip of the leaves. Values encircled in green are explicitly given as premises by the authors, while other values are inferred.

MST originally modeled the plant vascular system as a widened pipe model (WPM), wherein conduits widen from tip to base (2, 3, 5) (Fig. 1*A*). A decade later, MST adopted a coalescing vascular model (4) which allows conduits to merge along the conduit path from plant tip to base, to maintain conductive area fraction (Fig. 1*B*). Recent empirical observations (5–8) suggest a transition between the vascular models of West et al. (2) and Savage et al. (4). Rosell and Olson (8) detail how distinct transport and diffusive domains characterize plant vascular systems with a widened pipe model along the stem and a coalescing, possibly area-preserving model, at the end of the network (Fig. 1*C*). These domains were proposed after the findings of Lechthaler et al. in leaves (7) which is in line with the wider literature (2, 4, 9–11) and further supported within stems by Koçillari et al. who modeled conduit shape through the Pareto front of trade-off between carbon cost and hydraulic conductance (6). To date, the WPM is the leading theory explaining the shape of the vascular system (6). The empirical observation that conduit widening tip to base and conduit coalescence rates are variable along the network open up an avenue of investigation for the development of MST.

In this contribution, we deduce an MST-based model stemming from an energy efficiency premise alone, with a consequential reduction in total volume (Fig. 1*D*). Ours is a generalized MST model because it incorporates variable rates of conduit widening and coalescence, allowing predictions from the leaves in addition to stems. We investigate the implications of a vascular model of transitional functionality, from a transport (widened pipe model based) to a diffusive (coalescence based model) vascular network, building upon the framework of MST. We show that the premises of constant sapwood area across all branching levels and resistance maintenance are mutually exclusive. Furthermore, we demonstrate that if stem taper is variable, then the cumulative volume of branches in sequential branching generations cannot be preserved while maintaining an energy-efficient network. Thus, we challenge the notion of constant sapwood area tip-to-base (Fig. 1*D*) and argue that if resistance is to remain constant along the vascular network while conduit number increases, allowing for a diffusive domain in leaves for the efficient distribution of resources, then there must be a reduction in branch volume. Thus, vascular optimality influences the external plant morphology. We deduce relationships showing that this reduction in volume is necessarily commensurate with the increase in coalescence rate in leaves. Last, we detail how carbon investment is comparable or lower than previous MST models due to it being offset by reduced volume. In the discussion, we consider the implications for the carbon investment needed for plant growth and the potential use of our model to investigate causes of differential tree mortality.

## Theory Development and Results

**Fig. 2.**
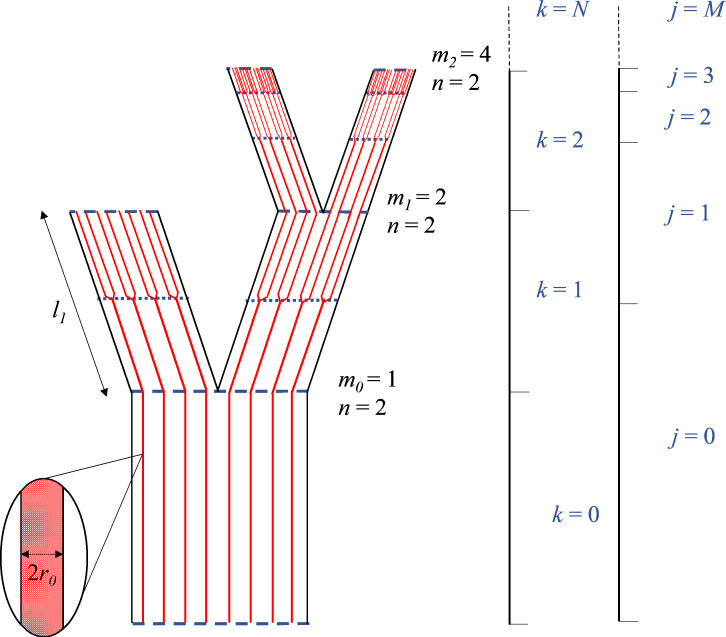
Illustration of a vascular network with generations of branching k = 0 to 2 and conduit coalescence/furcation j= 0 to 3. Conduits are shown in red, whereas the external branching network is shown in black. The branching locations are denoted with horizontal blue dashed lines, whereas the blue dotted lines denote conduit coalescence/furcation nodes. Example values for $m_{k}$ and n are given across each branching generation, whereby $m_{k}$ increases with coalescence rate at higher generations.

MST describes a set of scaling relationships that model branch dimensions across branching generations (2, 4). The hydraulic architecture of vascular plants can be characterized through branching ratio (n), conduit radii ($r_{k}$) and length ($l_{k}$) (2, 3), and conduit coalescence ratio ($m_{k}$) (4) at any given branching generation (k=0,1,...,N) (Fig. 2). In the following sections we deduce generalized MST relationships for a) the area of conduits ($r_{k}$) and their total summed area ($R_{k}$), b) the length of branches ($l_{k}$) in association with their volume, and c) an energy efficient system that maintains hydraulic resistance ($\omega_{k}$) along the network. We deduce that the premise of energy efficiency alone can be used to derive MST relationships, and moreover show how d) it determines the overall volume of the plant, and elaborate on e) the relationships that dictate the transition from a transport domain to diffusive domain and f) the associated overall carbon cost. Finally, using data from Koçillari et al. (6) we show g) empirical support for aspects of our generalized MST model.

### A. Conductive Area Preservation/Conduit Radii.

Here, we describe the relationships pertaining to conduit area and its widening along generations. West et al. (3) included a widening term ($t_{R}$) in the MST relationships ($r_{k}^{2}=n^{t_{R}}\cdot r_{k+1}^{2}$), for which $t_{R}$ is the rate of change between $r_{k}^{2}/r_{k+1}^{2}$ and n on a logarithmic scale assuming a pipe model that widens toward the base ($m_{k}$=1) (6). Allowing $m_{k}$ to vary creates a more generalized relationship:

[1] $$\begin{array}{c} \frac{r_{k}^{2}}{m_{k}\cdot r_{k+1}^{2}}=\frac{R_{k}^{2}}{R_{k+1}^{2}}=n^{t_{R}}, \end{array}$$

where $R_{k}$ is the radius of a total volume containing all the conduits at the base of the branch k (red volume in Fig. 1). Thus, $t_{R}$ is the rate of change between $R_{k}^{2}/R_{k+1}^{2}$ and n on a logarithmic scale for any value of $m_{k}$. There is a relationship between the branching ratio n and conduit coalescence $m_{k}$, such that $m_{k}=n^{p_{k}}$, where $p_{k}\geq 0$ (4). A relationship for individual conduits can thus be rewritten as:

[2] $$\begin{array}{c} \frac{r_{k}^{2}}{r_{k+1}^{2}}=n^{\left(t_{R}+p_{k}\right)}=n^{t_{r}}, \end{array}$$

where $t_{r}$ is the rate of change between $r_{k}^{2}/r_{k+1}^{2}$ and n on a logarithmic scale under the assumption that conduits coalesce ($m_{k}\neq 1$). Hence, whenever there is conduit coalescence, we shall make a difference between $t_{r}$ and $t_{R}$ (4), under the equality $t_{r}=t_{R}+p_{k}$ that derives from Eq. **2**. Some authors suggest that total conductive fraction may be preserved along branching generations ($r_{k}^{2}=n\cdot r_{k+1}^{2}$ within a volume filling network) (4, 11), and the model of Savage et al. (4) allowed the conditions for which $t_{R}=0$ while a given conduit widening rate may still exist (these conditions would be $p_{k}=1$ and $m_{k}=n$). Further work estimated species-specific values for these traits (12, 13). Recent empirical observations suggest that there may be a part of the plant for which those conditions are met, but not along all branching generations (7), and thus we explicitly made m and p variable according to k (Fig. 2). Thus, a $t_{r}$ value between 0 and 1, captures the change in conduit area along branching generations ($r_{k}^{2}=n^{t_{r}}\cdot r_{k+1}^{2}$) by including both widening and coalescence. The recent experimental data (6–8) can be modeled through Eq. **2** whereby their findings suggest a transition from $t_{r}=1/6$ at the plant base to $t_{r}=1$ at the plant tip (within a volume filling network), coinciding with the two different models suggested by West et al. (3) and Savage et al. (4), respectively (Fig. 1).

### B. Volume Preservation/Branch Length.

Here, we describe the relationships pertaining to branch volume and its tapering along generations. The lengths of conduits within any given branching generation can be modeled as equivalent to the length of that segment, and their cubic power proportional to its volume. Thus, $l_{k}^{3}$ is described through the term “service volume” referring to the volume of cells supported by a branch of a given size (2). MST’s original formulation gave a relationship between sequential branch lengths that relied upon branch volume preservation, which lead to the deduced allometric scaling (3). Empirical observations however indicate that this premise occurs inconsistently within natural systems (1). To remedy this, a branch segment taper term ($t_{l}$) can be included in the original MST formulation (1):

[3] $$\begin{array}{c} \frac{l_{k}^{3}}{l_{k+1}^{3}}=n^{\left(1/t_{l}\right)}, \end{array}$$

where branch length decreases $t_{l}$ ($t_{l}\leq 1$) gives the rate of change between $l_{k}^{3}/l_{k+1}^{3}$ and n on a logarithmic scale, whereby a value of $t_{l}=1$ results in branch volume preservation ($l_{k}^{3}=n\cdot l_{k+1}^{3}$) and $t_{l}<1$ brings a reduction in branch volume ($l_{k}^{3}=n^{1/t_{l}}\cdot l_{k+1}^{3}$). West et al. (3) use branch volume preservation $t_{l}=1$ as a premise of MST, and thus $t_{l}$ is a term not included in the original MST relationships. Like the widening of conduits tip to base, the empirical observations on the tapering of volume base to tip shows that it varies with relative length $t_{l}=f\left(l\right)$ (6), consequently decreasing along successive branching generations, and generating the values of $1/6<t_{l}<1$ as limiting boundary conditions.

### C. Resistance Maintenance (Energy Efficiency).

Here, we illustrate how resistance maintenance relates to conduit radii and branch volume widening/tapering terms. Eqs. **2** and **3** are integral to the assessment of hydraulic resistance along a branching system, which can be modeled through the Poiseulle formula that gives the hydraulic resistance of a conduit ($\omega_{k}$) as $8\mu l_{k}/r_{k}^{4}$, where μ is fluid viscosity (14). Resistance can be maintained along the entire vascular structure, which would minimize energy loss in the vascular system. West et al. (3) demonstrated this by comparing the total resistance along a single conduit (from plant base to plant tip) to that of the conduit resistance in the petiole, showing that a single conduit widening value could create an energy-efficient vascular architecture in a volume filling network, independent of path length. Therefore, within an optimal network, the resistance of a conduit in branch k is approximately equivalent to the resistance of a conduit in its daughter branch (k+1) ($\omega_{k}\approx \omega_{k+1}$). Alternatively, using Eqs. **2**–**3** which involve the empirical evidence that there is a variable widening rate in the radius $t_{r}$ and length $t_{l}$ of conduits, the maintenance of hydraulic resistance along generations can be assessed as:

[4] $$\begin{array}{c} \frac{\omega_{k}}{\omega_{k+1}}=\frac{n^{2t_{r}}}{n^{\left(1/3t_{l}\right)}}=n^{\left(2t_{r}-1/3t_{l}\right)}=1, \end{array}$$

whereby if resistance is maintained $2t_{r}-\frac{1}{3t_{l}}$ must equal 0. This allows resistance to be constant along a conduit path, for any values of n and k. As an alternative to West et al. (3), gMST works exclusively under the premise of hydraulic resistance preservation for energetic efficiency. Furthermore, it allows the fraction of conductive area to vary along the branching network, depending on both stem tapering base to tip and conduit widening tip to base ($t_{l}$ and $t_{r}$, see next section) and the exponent $p_{k}$ which regulates the coalescence rate, giving a more generalized MST relationship.

### D. Vascular Optimality Dictates Plant Morphology.

Here, we describe how resistance maintenance could influence plant morphology. The energy efficiency premise outlined implies that the transition from a transport domain to a diffusive domain can only be yielded by a reduction in branch volume, thus altering plant morphology (Fig. 1*D*). This transition occurs because the increase in coalescence needed for a diffusive domain must be accompanied by stem tapering if resistance is to remain constant over the vascular network (Fig. 2). If resistance maintenance and the need for an efficient diffusive network are both factors determining natural selection, then this reduction in volume demonstrates how vascular optimality impacts morphology. Eq. **4** leads us to deduce that there is a constant relationship between the widening and coalescence of conduits tip to base and the tapering of branch volumes base to tip, that describes the change in morphology:

[5] $$\begin{array}{c} t_{r}=\frac{1}{6t_{l}}. \end{array}$$

Hence, as the widening of conduits has been experimentally observed to change along the network (6–8), then volume need not be preserved, meaning resistance preservation could solely regulate the vascular system. In (Fig. 1*D*), we show that given the relationship deduced in Eq. **5**, the model of total conduit volume observed by Rosell and Olson (8) necessarily leads to an ensuing model of plant morphology that modifies the total volume accordingly. In other words, as conduit widening tip to base has been observed to decrease along branching generations, the tapering of the total volume of successive branching generations must increase, impacting the MST scaling.

### E. Transition from Transport to Diffusive Network.

Here, we describe how the network transitions between transport and diffusive domains along the length of conduits, showing where along the plant’s length this functional transition occurs. The internal vascular system can coalesce independently from the exterior branching network, and thus the generations of conduits j and branches k operate at different scales (Fig. 2). All equations can be calculated for j as for or k, bringing different values of widening for the internal vascular network which we denote with a tilde $\tilde{t_{l}}$ (Fig. 1*D*). The internal vascular network can be modeled independently of the external branching nodes using the MST relationships:

[6] $$\begin{array}{c} \frac{l_{j}^{3}}{l_{j+1}^{3}}=n^{\left(1/{\tilde{t}}_{l}\right)}, \end{array}$$

where $l_{j}$ represents the length of a conduit generation j between coalescence nodes and $\tilde{t_{l}}$ gives the rate of change between $l_{j}^{3}/l_{j+1}^{3}$ on a logarithmic scale (Fig. 2). It is possible to deduce at what distance from the stem tip that the functional transition between a transport and diffusive vascular network occurs, by describing it through the summation of an infinitely scaling geometric series.

[7] $$\begin{array}{c} l_{t}=\frac{l_{j=0}}{1-n^{\left(-1/3\tilde{t_{l}}\right)}}, \end{array}$$

where $l_{t}$ represents total network length, and $l_{j=0}$ denotes the length of the basalmost conduit through the branching system until its first branching node (j=0) (Fig. 2). The value of $\tilde{t_{l}}$ varies inversely with stem volumetric taper, exhibited within Fig. 1*D* by the inverse relationship between the external branching and the internal conductive volume. $\tilde{t_{l}}$ has a value of 1/6 at the base of the stem, which implies $l_{j=0}=3/4\cdot l_{t}$ (*SI Appendix*, S1). It is thus plausible that approximately the first three-quarters of the height of a tree from its base has a transport function, with no conduit coalescence, whereas in the topmost quarter the vascular network transitions into an increasingly diffusive function with an exponential increase in coalescence. Our estimates would therefore imply about 3 to 4 coalescence nodes located within twigs, with all other conduit branching occurring within the leaves. Coalescence rates are therefore modeled from the tip of the leaves to the base of the stem at the vascular nodal scale, and thus all observations, whether in the stem or leaves can be modeled within our gMST model.

### F. Carbon Economy.

Finally, we quantify the effect on the carbon cost ($C_{c}$) of the vascular system, in response to Eqs. **1**–**5**. $C_{c}$ is often defined as the total volumetric investment in the vessel walls, whereby vessel wall thickness is expected to be proportional to vessel area. $C_{c}$ can be quantified through the summation of a geometric series:

[8] $$\begin{array}{c} C_{c}\propto \frac{r_{k=0}^{2}l_{k=0}}{1-n^{-t_{R}}n^{\left(-1/3t_{l}\right)}}. \end{array}$$

**Fig. 3.**
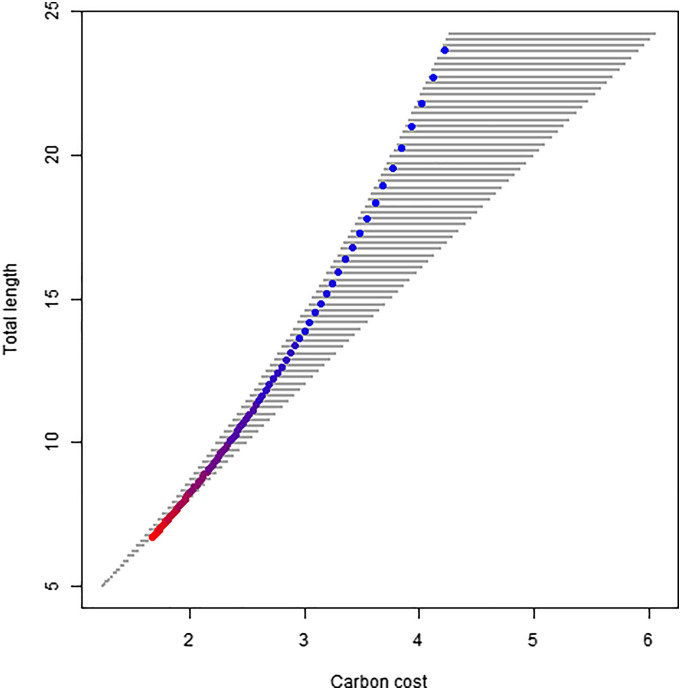
Results of a Monte-Carlo simulation combining values between 0 and 1/6 for $t_{R}$ and 0 and 1 for $t_{l}$, showing the resultant effect upon carbon cost and total plant length. The colored points indicate the predicted model from Eq. **5** with the color indicating the plant average values of conduit coalescence ratio relative to branching ratio $\bar{p_{k}}$ increasing from 0 (blue) to 1 (red).

Using Eq. **8**, the carbon cost of the widened pipe model (3) can be calculated using the values $t_{l}=1$ and $t_{R}=1/6$. For the coalescence model (4), it can be ascertained with the values of $t_{l}=1$ and $t_{R}=0$. The carbon cost of our gMST model can be predicted with an approximate average value slightly lower than $t_{R}<1/6$ (8) as Eq. **5** can be substituted in to Eq. **8**. For a given length and basal diameter of the first segment, $r_{k=0}$ and $l_{k=0}$, our generalized MST model would result in a total carbon expenditure systematically lower than either West et al.’s (3) or Savage et al.’s (4) models (all calculations can be found as *SI Appendix*, S2). This carbon efficiency in our model is achieved through the reduction of volume. However, this reduction occurs at the expense of height when considering k=0 dimensions. When comparing the carbon cost of individuals of equal height our model proves to approach that of West et al. (3), seen within Fig. 3. Selection must therefore balance the need to transition into a diffusive network with carbon cost, for plants of equivalent height, assuming that resistance remains constant.

To investigate the carbon investment that results from following different strategies in the morphology of the vascular network, we conducted a Monte-Carlo (MC) simulation combining varying values of $t_{R}$ and $t_{l}$ (widening of conduits tip to base and tapering of volume base to tip respectively) to illustrate their effect upon total length and carbon cost. The result is given in Fig. 3, with the gray points indicating the MC simulation results, generating a region of plausible plant length and carbon cost values. Our hydraulically optimal equation derived above (Eq. **5**) is shown with varying values of $t_{R}$ and coalescence ratio relative to branching ratio ($p_{k}$, denoted as colored values in Fig. 3). The MC results demonstrate how coalescence rate and conduit widening influence our gMST model, with increasing values of $p_{k}$ being associated with lower total length and carbon cost for the same hydraulic performance. A lower rate of conduit taper attained by increased conduit coalescence means that plants can grow taller, while maintaining hydraulic optimality with a lower carbon expenditure. Thus, Eq. **5** is a highly carbon-efficient strategy that allows plants to maintain diffusive functionality while growing taller.

### G. Empirical Support and Comparison against Previous MST Models.

Our generalized MST model was tested against empirical data including its implications for vessel frequency and conduit area along the length of the plant. We compared simulated values from our model and previous MST models to data available in Koçillari et al. (6). These data included both the stems and sometimes leaves of a wide range of vascular plants, thus allowing us to model the vast majority of the vascular network under our MST presumptions (Eqs. **1**–**7**) against empirical data.

**Fig. 4.**
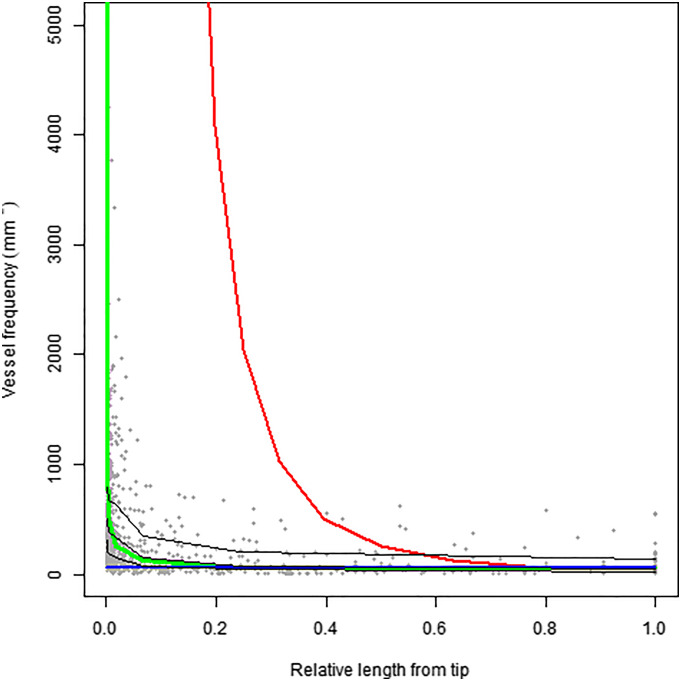
Plot of relative plant length vs. vessel frequency. Three models are plotted, with our generalized MST model given in green, West et al.’s (3) model given in blue, and Savage et al.’s (4) model given in red. The black lines represent the 75th, 50th, and 25th quantiles for the dataset. Relative length is given by the distance from the tip divided by the total length of the plant.

**Fig. 5.**
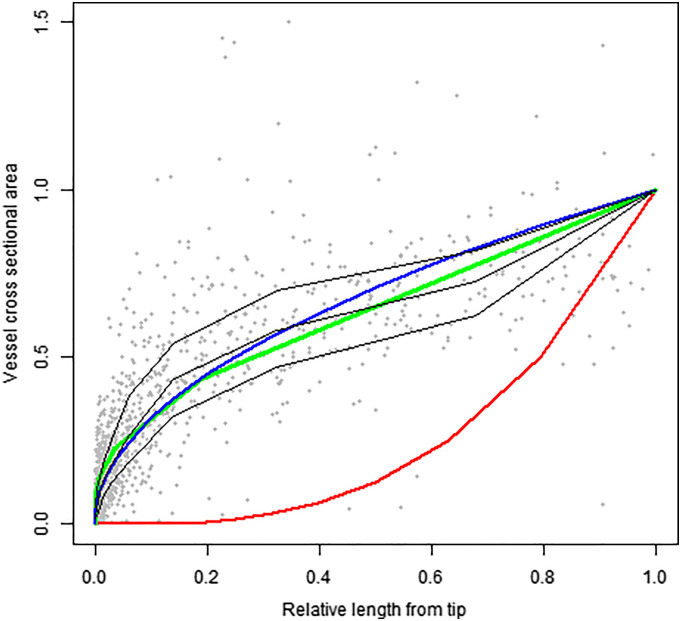
Plot of normalized plant vessel cross-sectional area vs. relative length. Three models are plotted, with our generalized MST model given in green, West et al.’s (3) model given in blue, Savage et al.’s (4) model given in red. The black lines represent the 75th, 50th, and 25th quantiles for the dataset. Relative length is given by the distance from the tip divided by the total length of the plant.

Figs. 4 and 5 show the simulated value for our generalized MST model (in green) against empirical data, showing as well the MST models of West et al. (3) (in blue) and Savage et al. (4) (in red). The results illustrate that the generalized MST model accurately models vessel frequency (Fig. 4) and cross-sectional area (Fig. 5) stretching from the stem to the leaves, compared to other MST models. It should however be noted that previously proposed MST models were intended to model only the stems, i.e., excluding the leaves, but still predicted rampant furcation.

Our generalized MST model outperformed other models in predicting empirical observations on vessel frequency (Fig. 4). West et al.’s (3) model proved to perform well for the majority of the vascular network, although its lack of conduit coalescence results in poor performance in the latter stages of the network (i.e., toward the leaves). Savage et al.’s (4) model yielded a rapid increase in vessel frequency, similar to our model, although at an earlier stage of the network. Kolmogorov–Smirnov tests showed that our generalized MST model predictions of vessel frequencies were the ones that fit best (*D* = 0.571; *P-value* = 0.141), since the predictions of West et al. (3) (*D* = 0.777; *P-value* = 0.009) and Savage et al. (4) (*D* = 0.714; *P-value* = 0.021) could be proven to significantly differ from the empirical observations. Our generalized internal MST vascular model therefore combines aspects of both models to successfully predict vessel frequency with relative length.

Second, we modeled how our model predicts vessel cross-sectional area, by simulating vessel cross-sectional area against distance from the tip (Fig. 5). In this comparison, only the predictions from Savage et al.’s (4) model significantly differed from the distribution of the empirical data (*D* = 0.7; *P-value* = 0.0123). Strikingly, the results showed that our modeled rate of widening alongside conduit coalescence yields a similar cross-sectional area as West et al.’s (3) assumptions of fixed conduit widening with no coalescence. We therefore agree with past suggestions that lumen resistance scales with a number of factors such as pit membrane resistance and vessel/tracheid length (6). Our vascular equations (Eqs. **6** and **7**) are applicable within both leaves and stems, and therefore they attain an gMST model applicable for the entire plant vascular system, i.e., including the leaves which were not included in previous MST formulation (3).

## Discussion

Rosell and Olson (8) describe the hydraulic architecture of trees as having a “transport” and “diffuse” domain within their vascular networks. The branching system can therefore both transport resources to the farthest parts of the organism and then transition to the distribution of these resources to the tissues where they are needed. Our set of generalized MST relationships (Eqs. **1**–**5**) give the relationship between $t_{r}$ and $t_{l}$ which allows this transition between the models of West et al. (3) and Savage et al. (4). The premise that “service volume” would be constant ($t_{l}=1$) yields a constant value for the widening rate of individual conduits $t_{r}=1/6$ (Eq. **5**), which minimizes resistance for the transportation of resources without losing volume and is thus congruent with West et al.’s (3) models and empirical observations at the beginning of the network (6–8). However, when approaching the leaves and within them, $t_{r}$ must approach one for conduit area preservation while resistance remains constant and branch volume decreases. Maintaining constant resistance with a $t_{r}$ value of one is facilitated by increasing $m_{k}$, which maximizes conductance and resource distribution (4, 9). As a consequence, $t_{l}$ must become substantially lower than one, meaning volume is increasingly lost in the terminal generations. This gives a gMST model of plant morphology (Fig. 1*D*) deduced from the resistance maintenance premise alone (Eq. **4**), given the variable widening and coalescence rates observed in recent research (6–8). The implications may in fact reach beyond just plants, with possible insights into the morphology of other taxonomical groups (e.g., if animals have larger shares of diffusive functionality in their vascular network, a reduction in volume that modifies their allometric scaling would be implied). Further empirical research in a wide range of fields could be stimulated by our MST modeling based on energy efficiency, such as our predictions of conduit coalescence locations (and lack thereof), or the predicted rates of conduit coalescence and volume reduction within branching generations.

The biologically optimal resource distribution network is underpinned by Eq. **5**. Natural selection should favor vascular structures that maintain hydraulic resistance while increasing conduit number at the network’s termination. Increasing conduit coalescence rate must therefore be compensated for by the reduction in external volume. Thus, our theory agrees with previous postulates challenging Leonardo’s rule of area preservation (1), giving a unified gMST framework that can accommodate transport and diffusive domains within the plant.

The transition from a transport to a diffusive domain can occur while maintaining a constant level of resistance. This is however achieved through different mechanisms, with resistance maintenance being generated by widening ($t_{r}$) in the transport domain and conduit coalescence ($m_{k}$) in the diffusive domain. The implication of this difference is that if selection favors resistance maintenance and some degree of diffusion in the network, volume cannot be maintained with respect to conduit area (Eq. **5**). Trees offer an ideal demonstration of this trade off between volume and vascular diffusion, with conduits in the trunk and the first few branching generations coming close to volume preservation, as long-distance transportation is favored. A transition to a diffuse domain occurs within the later branching generations (which we calculated to occur within the last quarter of the total length of the network), allowing resources to be distributed efficiently within leaves, and thus tree volume is reduced leading to the termination of the branching network. Such a transition is in line with currently available data (6–8), and our results demonstrated that incorporating a transition between a transport and diffusive domain into MST modeling through variable widening and conduit coalescence achieved a better empirical fit to the entire vascular network including both leaves and stems (Figs. 4 and 5). Our prediction is however based on a constant conduit widening rate that may not always be the case. It is possible that the first conduit branching location occurs closer to the tip than estimated, in which case plant morphology may be more acutely impacted by changes in widening rate (Eqs. **2** and **5**). Future research is therefore needed to obtain empirical data to evaluate the rate of conduit coalescence within branches.

The carbon cost of our gMST model proved to be highly comparable to that of West et al. (3) for equivalent plant lengths. However, the height and carbon cost of the plant changes with respect to $t_{l}$, and thus with respect to conduit coalescence and widening rates. Consequently, the carbon cost is lower than all previous MST models if stem volumetric tapering occurs. This implies that the resistance maintenance premise may result in scaling exponents lower than predicted by area/volume preservation (15). Moreover, the coalescence model (4) predicts an enormous increase in the number of conduits, diverging from empirical observations and leading to much higher carbon costs than the widened pipe model (3). Our model therefore predicts that natural selection will favor individuals that invest less carbon in the transport and diffusive domains for the same performance, giving more carbon surplus that can be invested in other plant functions like reproduction or growth. Our generalized MST model introduces the same conduit coalescence concepts as Savage et al. (4), but in such a way that does not imply a massive increase in carbon cost, effectively merging the coalescence and the widened pipe model, and better predicting the number of conduits per unit leaf area along the length of a plant (Fig. 4). Fig. 3 demonstrates the impact of coalescence upon carbon cost, where the relationship of Eq. **5** is highlighted in color, illustrating that it provides a path of carbon cost reduction for any given total length of the vascular network. Selection will favor networks that minimize carbon expenditure while also maintaining resistance across the vascular network as plants grow taller. Selection should thus favor the scenario presented here, with a gradual transition to a diffusive domain at the termination of the network, thus minimizing excess carbon expenditure while keeping constant hydraulic resistance constant, through the reduction of plant volume. The carbon cost for otherwise equivalent performance proves to be marginally more than of West et al. (3), but more accurately depicts the plant vascular system.

The covariation of MST exponents has been examined empirically by previous authors (16, 17), but no rigorous theory-based reasoning has been put forward to describe the observed covariation. In this contribution, we offer a mechanistic theory relating widening/tapering coefficients in Eqs. **4** and **5**, which may offer an explanation for this covariation. If hydraulic resistance maintenance and carbon cost are key factors of selection, then the covariation of MST exponents can be derived from the rate of conduit coalescence and widening. Furthermore, branching of the internal vascular network can be independent of branching of the exterior branching nodes. Savage et al. (4) partially segregated the internal and external networks by modeling widening and coalescence within the plant vascular system, but maintained that the proportion of conduit area was a fixed proportion of the stem area (Fig. 1*B*). We complete this network segregation and illustrate how energy efficiency dictates variation in coalescence rate through the plant (Eqs. **6** and **7**). This way the plant can show phenotypical plasticity to environmental circumstances, such as water availability, thus influencing the height and morphology of the plant in connection with its hydraulic architecture.

We reconsider MST in the light of recent empirical observations, outlining an MST-based model that accommodates for distinct transport and diffusive domains within a single plant vascular system and including the entire organism with both stems and leaves. Our model shows that natural selection should favor plants whose conduits coalesce and widen tip to base, compensated by an overall reduction in plant volume, maintaining hydraulic efficiency (Fig. 1*D*). We model how conduit coalescence could function within such a system, with the coalescence rate increasing dramatically in the final stages of the branching network, affecting the morphology of the plant and thus its efficient use of carbon. We therefore encourage authors to test both the premises and empirical strength of the proposed model and compare to other current models that explore the trade-offs between the vascular system and carbon cost, like that of Koçillari et al.’s WPM (6).

## Materials and Methods

In Section F on carbon economy, Monte-Carlo simulations were carried out with average stem and conduit taper and widening coefficient ($\bar{t_{l}}$ and $\bar{t_{R}}$) such that all coefficients could be applied in equations for total carbon cost and length. To model gMST within the Monte-Carlo simulation results, only plant average values of $\bar{t_{R}}$ and $\bar{p_{k}}$ were required and utilized to output a set of plant average values for $\bar{t_{r}}$ and $\bar{t_{l}}$. Thus, the total length and carbon cost could be calculated under our premise of energy efficiency (Eq. **5**).

In Section G, we compared different MST-based model predictions against empirical data kindly made available by Koçillari et al. (6). We used a Kolmogorov–Smirnov test to compare the predictions of each modeled conduit generation sequence against the observed sample frequency distributions. All calculations are available in the R scripts included as *SI Appendix*.

In order to simulate vessel frequency from our model, Eq. **6** was used to find the lengths of the first 100 j generations, using $l_{j=0}=0.75$ (an approximation deduced in *SI Appendix*, S1). $t_{l}$ was modeled to exponentially increase to a value of 1 toward the 100th iteration. For both our model and Savage et al.’s (4), we assumed that branching of conduits occurred at each node, and thus calculated the number of conduits in each generation starting from a single conduit, whereas for West et al. (2), it was assumed that no conduit coalescence occurs. The mean basal vessel frequency for all plants was then multiplied by the simulated vessel number for each generation to simulate vessel frequency along the distance of the stem.

We analyzed our model’s predictions of vessel cross-sectional area. Using the same assumptions as MST, we simulated the relative cross-sectional area for a conduit with a widening coefficient ($t_{R}$) that was fixed for West et al. (2) and Savage et al. (4), in accordance with Fig. 1, whereas for our generalized MST model, this value was allowed to vary. The widening coefficient was combined with the simulated internal node locations (calculated through Eqs. **6** and **7**).

## Supplementary Material

Appendix 01 (PDF)

Dataset S01 (CSV)

Dataset S02 (CSV)

## Data Availability

Previously published data were used for this work (6).

## References

[1] L. P. Bentley , An empirical assessment of tree branching networks and implications for plant allometric scaling models. Ecol. Lett. **16**, 1069–1078 (2013). 23800188 10.1111/ele.12127

[2] G. B. West, J. H. Brown, B. J. Enquist, A general model for the origin of allometric scaling laws in biology. Science **276**, 122–126 (1997). 9082983 10.1126/science.276.5309.122

[3] G. B. West, J. H. Brown, B. J. Enquist, A general model for the structure and allometry of plant vascular systems. Nature **400**, 664–667 (1999).

[4] V. M. Savage , Hydraulic trade-offs and space filling enable better predictions of vascular structure and function in plants. Proc. Natl. Acad. Sci. U.S.A. **107**, 22722–22727 (2010). 21149696 10.1073/pnas.1012194108 PMC3012458

[5] M. E. Olson, T. Anfodillo, S. M. Gleason, K. A. McCulloh, Tip-to-base xylem conduit widening as an adaptation: Causes, consequences, and empirical priorities. New Phytol. **229**, 1877–1893 (2021). 32984967 10.1111/nph.16961

[6] L. Koçillari , The Widened Pipe Model of plant hydraulic evolution. Proc. Natl. Acad. Sci. U.S.A. **118**, e2100314118 (2021). 34039710 10.1073/pnas.2100314118 PMC8179198

[7] S. Lechthaler, P. Colangeli, M. Gazzabin, T. Anfodillo, Axial anatomy of the leaf midrib provides new insights into the hydraulic architecture and cavitation patterns of Acer pseudoplatanus leaves. J. Exp. Botany **70**, 6195–6201 (2019). 31365742 10.1093/jxb/erz347 PMC6859715

[8] J. A. Rosell, M. E. Olson, To furcate or not to furcate: The dance between vessel number and diameter in leaves. J. Exp. Botany **70**, 5990–5993 (2019). 31738433 10.1093/jxb/erz362

[9] K. A. McCulloh, J. S. Sperry, F. R. Adler, Water transport in plants obeys Murray’s law. Nature **421**, 939–942 (2003). 12607000 10.1038/nature01444

[10] K. A. McCulloh, J. S. Sperry, F. R. Adler, Murray’s law and the hydraulic vs mechanical functioning of wood. Funct. Ecol. **18**, 931–938 (2004).

[11] J. S. Sperry, U. G. Hacke, J. Pittermann, Size and function in conifer tracheids and angiosperm vessels. Am. J. Botany **93**, 1490–1500 (2006). 21642096 10.3732/ajb.93.10.1490

[12] E. von Allmen , A species-level model for metabolic scaling of trees II. Testing in a ring- and diffuse-porous species. Funct. Ecol. **26**, 1066–1076 (2012).

[13] J. Sperry , A species-level model for metabolic scaling in trees I. Exploring boundaries to scaling space within and across species. Funct. Ecol. **26**, 1054–1065 (2012).

[14] M. H. Zimmermann, Xylem Structure and the Ascent of Sap (Springer, 1984), vol. 59, pp. 475–476.

[15] L. P. Bentley , An empirical assessment of tree branching networks and implications for plant allometric scaling models. Ecol. Lett. **16**, 1069–1078 (2013). 23800188 10.1111/ele.12127

[16] C. Price, B. Enquist, V. Savage, A general model for allometric covariation in botanical form and function. Proc. Natl. Acad. Sci. U.S.A. **104**, 13204–13209 (2007). 17664421 10.1073/pnas.0702242104 PMC1941814

[17] C. Price, B. Enquist, Scaling mass and morphology in leaves: An extension of the WBE model. Ecology **88**, 1132–41 (2007). 17536400 10.1890/06-1158

