# Supplementary material for: Vascular optimality dictates plant morphology away from Leonardo’s rule
Source: Proc Natl Acad Sci U S A. 2023 Sep 18;120(39):e2215047120. doi: 10.1073/pnas.2215047120 (PMC10523467; doi:10.1073/pnas.2215047120)
Supplement: Supplementary file 1 — Appendix 01 (PDF) [file pnas.2215047120.sapp.pdf]

Supplementary Information for the paper titled "Vascular optimality dictates plant morphology away from Leonardo's rule"

September 11, 2023

S1. Calculating the transition between transportive and diffusive stages of vascular network

A plant can be modelled through two interconnected branching networks: the external supportive network and the internal vascular network. Many of the equation forms presented within the manuscript can given in relation to either of these networks with the appropriate altering of notation. Eq. 3 within the manuscript can be written as the following when considering the vascular network of conduits, using j to denote the branching generations along the vascular network, and $\tilde{t}_{lj}$ to denote the volumetric tapering of conduits.

$$\frac{l_j^3}{l_{j+1}^3} = n^{\left(\frac{1}{\tilde{t}_{lj}}\right)}. \tag{S.1}$$

Consequentially the above equation can be used to give the total length of the network, as has been done in the past while focusing on the external network West *et al.* (1). The below equation gives a rearrangement of the geometric summation to yield the length of the basalmost conduit $l_{j=0}$

$$l_{j=0} = l_t \cdot \left(1 - 2^{\left(-\frac{1}{3\cdot\tilde{t}_{lj}}\right)}\right). \tag{S.2}$$

Using the same mathematical development within the main manuscript, but for the internal network, the value of $\tilde{t}_{lj}$ is expected to be 1/6 at the base of the plant, assuming that hydraulic preservation underpins plant development. The value of $l_{j=0}$ can therefore be estimated:

$$7.5 = 10 \cdot \left(1 - 2^{\left(-\frac{1}{3\cdot 0.167}\right)}\right), \tag{S.3}$$

The above equation gives an example of a tree of $l_t = 10$, and values for other tree sizes will vary only slightly in terms of the proportion between transportive

and diffusive functionality. All in all, it shows that the length of the basalmost conduit could account for approximately 75% of the stems length, before conduit branching. This is therefore a curious aspect of the developed modelling framework, given in the main manuscript, that other authors could test empirically.

S2. Carbon cost calculations

The total carbon expenditure can be compared between models, using arbitrary values of $r_{k=0}$ and $l_{k=0}$ which represent the radii of a conduit and length of a branch in generation $k = 0$. The carbon cost of the widened pipe model proposed by West *et al.* (1) can be found through using the values $t_l = 1$ and $t_R = 1/6$. The carbon cost of Savage *et al.*'s (2) can be ascertained with the values of $t_l = 1$ and $t_R = 0$. Lastly, the carbon cost of the gMST model can be predicted with a value for t_R ($t_R < 1/6$), as Eq. 5 can be substituted into Eq. 8. Since gMST proposes a variable value for t_R along the length of the plant, its result depends on the average t_R value used. The average is expected to be somehow below 1/6, as $t_R = 1/6$ is a good approximation along the majority of the stem (3), with expected lower values towards the termination of the network (4), and thus we assumed a value of $t_R = 1/5$ for gMST. Using the arbitrary values of 0.5 m for r_0 and 5 m for l_0, we found that West *et al.*'s (1) model obtained $C_c \propto 4.3$, and Savage *et al.*'s (2) was $C_c \propto 6.1$, both obtaining higher carbon expenditure than the gMST model result which was $C_c \propto 3.7$. These calculations are as follows:
West *et al.* (1):

$$C_c \propto \frac{0.5^2 \cdot 5}{1 - 2^{-1/6} \cdot 2^{(-1/3)}} \propto 4.3; \tag{S.4}$$

Savage *et al.* (2):

$$C_c \propto \frac{0.5^2 \cdot 5}{1 - 2^0 \cdot 2^{(-1/3)}} \propto 6.1; \tag{S.5}$$

gMST:

$$C_c \propto \frac{0.5^2 \cdot 5}{1 - 2^{-1/5} \cdot 2^{(-1/2.5)}} \propto 3.7. \tag{S.6}$$

References

[1] Geoffrey B. West, James H. Brown, and Brian J. Enquist. A general model for the structure and allometry of plant vascular systems. *Nature*, 400(6745):664–667, 1999.

[2] V. M. Savage, L. P. Bentley, B. J. Enquist, J. S. Sperry, D. D. Smith, P. B. Reich, and E. I. Von Allmen. Hydraulic trade-offs and space filling enable better predictions of vascular structure and function in plants. *Proceedings of the National Academy of Sciences of the United States of America*, 107(52):22722–22727, 2010.

[3] Loren Koçillari, Mark E. Olson, Samir Suweis, Rodrigo P. Rocha, Alberto Lovison, Franco Cardin, Todd E. Dawson, Alberto Echeverría, Alex Fajardo, Silvia Lechthaler, Cecilia Martínez-Pérez, Carmen Regina Marcati, Kuo Fang Chung, Julieta A. Rosell, Alí Segovia-Rivas, Cameron B. Williams, Emilio Petrone-Mendoza, Andrea Rinaldo, Tommaso Anfodillo, Jayanth R. Banavar, and Amos Maritan. The Widened Pipe Model of plant hydraulic evolution. *Proceedings of the National Academy of Sciences of the United States of America*, 118(22), 2021.

[4] Julieta A. Rosell and Mark E. Olson. To furcate or not to furcate: The dance between vessel number and diameter in leaves. *Journal of Experimental Botany*, 70(21):5990–5993, 2019.
